# The Predictive Value of Risk Factors and Prognostic Scores in Hospitalized COVID-19 Patients

**DOI:** 10.3390/diagnostics13162653

**Published:** 2023-08-11

**Authors:** Milica Brajkovic, Miodrag Vukcevic, Sofija Nikolic, Marija Dukic, Marija Brankovic, Ana Sekulic, Viseslav Popadic, Mihailo Stjepanovic, Aleksandra Radojevic, Ljiljana Markovic-Denic, Nina Rajovic, Natasa Milic, Srdjan Tanasilovic, Zoran Todorovic, Marija Zdravkovic

**Affiliations:** 1 Clinic for Internal Medicine, University Clinical Hospital Center Bezanijska Kosa, 11080 Belgrade, Serbia; brajkovic.milica@yahoo.com (M.B.); nikolic.sofija94@gmail.com (S.N.); marijadukic3@outlook.com (M.D.); manive23@gmail.com (M.B.); sekulic.ana@bkosa.edu.rs (A.S.); viseslavpopadic@gmail.com (V.P.); radaleksandra@gmail.com (A.R.); markovic.denic@gmail.com (L.M.-D.); zoran.tdrvc@gmail.com (Z.T.); 2 Department of Pulmonology, University Clinical Hospital Center Zemun, 11080 Belgrade, Serbia; mikivukcevic@hotmail.com; 3 Faculty of Medicine, University of Belgrade, 11080 Belgrade, Serbia; mihailostjepanovic@gmail.com (M.S.); srdjantanasilovic@kcs.ac.rs (S.T.); 4 Clinic of Pulmology, Clinical Center of Serbia, 11000 Belgrade, Serbia; 5 Institute for Medical Statistics and Informatics, Faculty of Medicine, University of Belgrade, 11080 Belgrade, Serbia; nina94rajovic@gmail.com (N.R.); silly_stat@yahoo.com (N.M.); 6 Department of Internal Medicine, Division of Nephrology and Hypertension, Mayo Clinic, Rochester, MN 55905, USA; 7 Clinic of Dermatovenerology, Clinical Center of Serbia, 11000 Belgrade, Serbia

**Keywords:** score, intensive care, COVID-19, mortality

## Abstract

Introduction: Risk stratification in patients with COVID-19 is a challenging task. Early warning scores (EWSs) are commonly used tools in the initial assessment of critical patients. However, their utility in patients with COVID-19 is still undetermined. Aim: This study aimed to discover the most valuable predictive model among existing EWSs for ICU admissions and mortality in COVID-19 patients. Materials and methods: This was a single-center cohort study that included 3608 COVID-19 patients admitted to the University Clinical Hospital Center Bezanijska Kosa, Belgrade, Serbia, between 23 June 2020, and 14 April 2021. Various demographic, laboratory, and clinical data were collected to calculate several EWSs and determine their efficacy. For all 3608 patients, five EWSs were calculated (MEWS, NEWS, NEWS2, REMS, and qSOFA). Model discrimination performance was tested using sensitivity, specificity, and positive and negative predictive values. C statistic, representing the area under the receiver operating characteristic (ROC) curve, was used for the overall assessment of the predictive model. Results: Among the evaluated prediction scores for 3068 patients with COVID-19, REMS demonstrated the highest diagnostic performance with the sensitivity, PPV, specificity, and NPV of 72.1%, 20.6%, 74.9%, and 96.8%, respectively. In the multivariate logistic regression analysis, aside from REMS, age (*p* < 0.001), higher CT score (*p* < 0.001), higher values of urea (*p* < 0.001), and the presence of bacterial superinfection (*p* < 0.001) were significant predictors of mortality. Conclusions: Among all evaluated EWSs to predict mortality and ICU admission in COVID-19 patients, the REMS score demonstrated the highest efficacy.

## 1. Introduction

COVID-19 is a highly contagious disease caused by the SARS-CoV-2 virus. SARS-CoV-2 is easily spread and can cause a wide specter of diseases, from asymptomatic to acute respiratory failure and death. During the pandemic, healthcare workers face many challenges including recognition of those patients in greatest need of medical attention [1].

As a consequence of the large number of in-hospital patients with COVID-19 and limited health resources, the most important objective in clinical practice is to find the appropriate scoring system to evaluate those at higher risk of complications and lethal outcomes. There are many predictive factors including demographic, radiographic and laboratory parameters, but only a few of them can help in the early identification of patients at risk. Until now, it is demonstrated that cytokine regulation, inflammatory response, and micro- and macro-thromboembolic complications are the main pathophysiological mechanisms involved in poor clinical outcomes of patients with COVID-19 [2,3].

There is a complex network of regulatory mechanisms with an assignment to balance the production of pro-inflammatory and anti-inflammatory cytokines so that the reaction remains limited and sufficient for pathogenic noxa. The failure of one or more of these mechanisms can induce immune system overactivation and massive production of cytokines initiating a systemic inflammatory reaction with harmful consequences, which is widely known as a cytokine storm. A cytokine storm mainly displays as an influenza-like syndrome that may evolve or be complicated by multi-organ damage. For example, tachypnea and hypoxemia as symptoms are often present and can evolve into acute respiratory distress syndrome (ARDS), which is one of the most dangerous complications of COVID-19 infection [4]. Most deaths from COVID-19 ARDS have evidence of thrombotic disseminated intravascular coagulation. Coagulation dysfunction is regularly seen in COVID-19 and is detected by elevated D-dimer levels. Sadly, many cases with coagulation dysfunction have fatal outcomes where diffuse microvascular thrombosis is seen, suggesting thrombotic microangiopathy [5,6].

Having in mind the unpredictability of the disease and various pathophysiological mechanisms involved, making a proper risk stratification tool is a challenging task. Over time, various scores have been developed that are routinely used in community-acquired pneumonia and other conditions. Certain studies evaluated the diagnostic performance of these early warning scores (EWSs) in patients with COVID-19 but with controversial results. Martin-Rodriguez et al. demonstrated the best predictive capacity of NEWS2 early warning score, where patients with the score equal to or greater than 8 points had a high risk of clinical deterioration and a very high risk of two-day mortality [7]. The other study by Tsai et al. indicated that REMS score was superior to NEWS and MEWS for predicting the in-hospital mortality of COVID-19 patients [8]. In a study on 1501 patients, Veldhuis et al. reported that NEWS2 and the Quick COVID-19 Severity Index Score had the best diagnostic performance to predict ICU admission in COVID-19 patients [9]. As the COVID-19 pandemic progressed, the main focus was changed from more generic predictive scores to newly designed ones for patients with COVID-19 [10]. However, several predictive scores have been published, but only a few of them had been validated externally. Thus, the worldwide applicability of these prediction scores is still an unresolved question. All around the world, healthcare systems and patients profiles differ, which can impact these scores. Most of the scores have been validated in small populations, with a specific ethnic characterization, and do not appear to be effectively generalizable among different contexts [11].

Although there are no specific predictive scores for COVID-19, we mainly focused our research on early warning scores—EWSs. The EWSs were commonly used in an initial assessment of critical patients to help emergency physicians recognize those patients at greatest risk [2].

In this single-center study, we aimed to discover the most valuable predictive model among existing EWSs for ICU admissions and mortality in COVID-19 patients.

## 2. Materials and Methods

This is a single-center cohort study that included 3608 COVID-19 patients admitted to the University Clinical Hospital Center Bezanijska kosa, Belgrade, Serbia, between June 23, 2020 and April 14, 2021. The cohort included patients older than 18 years old with confirmed COVID-19 infection by positive real-time reverse-transcription polymerase chain reaction (RT-PCR) assay or antigen testing using nasal and pharyngeal swab specimens with clinical, radiographic or/and laboratory parameters that require hospitalization. The University Clinical Hospital Center Bezanijska kosa was one of the COVID-19 referent triage centers that treated more than 1000 patients per month and more than 100 patients in the ICU. Chest radiography (CXR) was performed on the day of admission and regularly controlled when needed. Chest CT to determine the severity of COVID-19 pneumonia was mandatory on admission day. The second control, CT chest, was indicated in patients with clinical signs of deterioration. The main criteria for ICU admission were radiographic, clinical and laboratory worsening, and oxygen saturation lower than 93% despite maximum oxygen support.

### 2.1. Data Collection

#### 2.1.1. Demographic, Anthropometric Data, Laboratory and Clinically Significant Parameters

The data were collected through medical documentation and the hospital’s health informational system (Heliant, v7.3, r48602). Demographic data (age, gender, and BMI), past medical history (hypertension, diabetes mellitus, COPD, coronary heart disease, heart failure, and chronic kidney disease), laboratory values (IL-6, CRP, PCT, ferritin, D-dimer, serum albumin, lymphocytes, thrombocytes, prothrombin time, activated partial thromboplastin time, and fibrinogen), and CT severity score were analyzed. Clinical and laboratory parameters were followed upon admission to the hospital (GCS, respiratory and hemodynamic parameters). These parameters were obtained by doctors or emergency registered nurses. Collected data were used to calculate prognostic scores (all variables included in the scores are presented in Supplemental Table S1).

#### 2.1.2. Prognostic Scores

For all 3608 patients, 5 early warning scores were calculated (MEWS, NEWS, NEWS2, REMS, and qSOFA). Parameters for every score, scoring system, and prognostic value are provided in Supplemental Table S1.

#### 2.1.3. COVID-19 Treatment

Patients were treated by the National Protocol of the Republic of Serbia for the treatment of COVID-19 infection and in concordance with the protocol suggested by the World Health Organization (WHO).

#### 2.1.4. Statistical Analysis

Numerical data were presented as mean with standard deviation or median with 25th and 75th percentiles. Categorical variables were summarized by absolute numbers with percentages. Differences between survivors and non-survivors were analyzed by Student’s *t*-test, Mann–Whitney U-test and Chi-square test for numerical and categorical data, respectively. Model discrimination performance was tested using sensitivity, specificity, and positive and negative predictive values. C statistic, representing the area under the receiver operating characteristic (ROC) curve, was used for an overall assessment of the predictive model. Univariate and multivariate logistic regression models were used to assess predictors of outcome as dependent variables. Significant variables from univariate analysis were included in multivariate regressions. Results were expressed as odds ratios (OR) and their corresponding 95% confidence intervals (CI). In all analyses, the significance level was set at 0.05. Statistical analysis was performed using IBM SPSS statistical software (SPSS for Windows, release 25.0, SPSS, Chicago, IL, USA).

#### 2.1.5. Ethics

The study was organized according to the principles of the Declaration of Helsinki of 1975, as revised in 2008 and approved by the Ethics Committee of the University Clinical Hospital Center “Bezanijska kosa”.

## 3. Results

### 3.1. Baseline Characteristics

A total of 3608 patients with COVID-19 infection were included in the study. Women (*p* = 0.035), older patients (*p* < 0.001), patients with comorbidities (*p* < 0.001), such as hypertension (*p* < 0.001), diabetes mellitus (*p* < 0.001), COPD (*p* = 0.003), coronary artery disease (*p* < 0.001), cardiomyopathy (*p* < 0.001) and malignancies (*p* < 0.001) more often had negative outcome. Sociodemographic characteristics and comorbidities of the study population according to outcome are presented in Table 1.

### 3.2. Radiographic Findings and Clinical Parameters of the Study Group according to Outcome

Patients more often had negative outcomes if they had bilateral pneumonia (*p* = 0.005), had a higher CT score (*p* < 0.001), were admitted to the ICU (*p* < 0.001), were on NIV/IMV (*p* < 0.001), had hospital-acquired pneumonia (*p* < 0.001), moreover ventilator-associated pneumonia (*p* < 0.001), had bacterial superinfection (*p* < 0.001), had ARDS (*p* < 0.001) or were on corticosteroid therapy (*p* < 0.001). Radiographic findings, need for oxygen support, hospital-acquired pneumonia and superinfection, ARDS and administrated therapy according to outcome are presented in Table 2.

### 3.3. Laboratory Parameters of the Study Group according to Outcome

Table 3 presents the laboratory parameters of the study population according to outcome.

### 3.4. Glasgow Coma Score, Pulmonary and Hemodynamic Parameters

Patients with Glasgow Coma Score <15 (*p* < 0.001), FiO2 at admission >21 (*p* < 0.001), increased number of respirations (*p* < 0.001), lower oxygen saturation (*p* < 0.001) and diastolic blood pressure (*p* < 0.001), lower MAP (*p* < 0.001) and higher heart rate (*p* < 0.001) more often had negative outcomes (Table 4).

### 3.5. Early Warning Scores (EWS) if the Study Group according to Outcome

Patients with higher MEWS (*p* < 0.001), NEWS (*p* < 0.001), NEWS2 (*p* < 0.001), REMS (*p* < 0.001) and qSOFA (*p* < 0.001) scores more often had negative outcome. Table 5 presents the prediction scores of the study population according to outcome. 

### 3.6. Diagnostic Performance of Early Warning Scores in the Study Group

The diagnostic performance of prediction scores used to predict mortality was tested. The sensitivity, PPV, specificity, and NPV of REMS were 72.1%, 20.6%, 74.9%, and 96.8%, respectively. Measures of diagnostics accuracy of other prediction scores are shown in Table 6.

**Figure 1:**
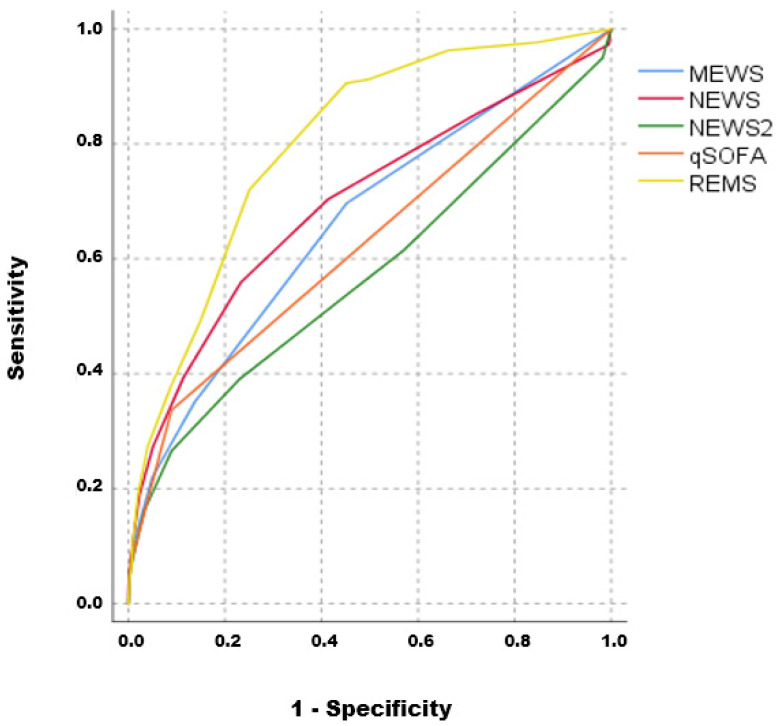
ROC analysis of prediction scores; AUC: MEWS = 0.662; NEWS = 0.695; NEWS2 = 0.572; REMS = 0.800; qSOFA = 0.626.

Based on the ROC curve analysis (Figure 1), the area under the curve (AUC) value for MEWS was 0.662, while for NEWS, it was 0.695, for NEWS2, it was 0.572, for REMS, it was 0.800 and for qSOFA, the AUC value was 0.626 (*p* < 0.001 for all). In Figure 1, an ROC analysis of the prediction scores is presented. 

### 3.7. Multivariate Logistic Regression Analysis

All variables significant in univariate logistic regression analysis were used in a multivariate model. Age (*p* < 0.001), higher CT score (*p* < 0.001), higher values of urea (*p* < 0.001), presence of bacterial superinfection (*p* < 0.001) and higher REMS score were significant independent predictors of mortality. Table 7 shows multivariate logistic regression analysis with outcome as a dependent variable. 

## 4. Discussion

In the present study, we determined the prognostic utility of different risk scores in predicting negative outcomes among COVID-19 patients. Between several identified predictors of mortality, age, CT score, urea levels, and the presence of bacterial superinfection were marked as the most significant in the multivariate logistic regression analysis. Regarding the predictive scores, REMS score was identified as the independent predictor of mortality, with the highest sensitivity and specificity among all evaluated scores (MEWS, NEWS, NEWS2, qSOFA).

Up to this point, several EWS models have been developed in predicting in-hospital mortality and the risk of ICU admission [12,13]. Although the significance of these scores has been proven in different conditions, the prognostic utility of these scores in patients with COVID-19 is yet to be determined.

MEWS is based on four physiological parameters and one observation—systolic blood pressure, heart rate, respiratory rate, temperature and APVU (Alert, Voice, Pain, Unresponsive) score [14]. A total score of 5 or more is likely to be associated with a higher incidence of admission to an intensive care unit or death. The advantage of MEWS over other scores, mainly SOFA and qSOFA, is the inclusion of temperature, respiratory rate, oxygen therapy, and oxygen saturation, which are important parameters in patients with COVID-19. Barnett et al. presented an adjusted MEWS score to predict in-hospital mortality in patients with COVID-19 by implementing additional respiratory parameters to increase sensitivity and specificity (CEWS) [15].

NEWS (National Early Warning Score) determines the degree of illness of a patient using six physiological findings and one observation [16]. In comparison with MEWS, it includes oxygen saturation and supplemental oxygen support. There is a lack of evidence regarding the significance of this score in COVID-19 patients, as the adjusted NEWS2 score was predominantly used in studies with COVID-19 patients [17].

NEWS2 (National Early Warning Score 2) is an updated version of the original NEWS. NEWS2 has incorporated parameters for respiratory insufficiency, involving the partial pressure of CO2, in comparison with the standard NEWS score [18]. Veldhuis et al. showed that NEWS2 ≥6 discriminated COVID-19 patients needing ICU admission with 78.1% sensitivity and 56.3% specificity, which is higher compared to our cohort, but with a significantly lower study sample size [9]. Although the predictive power of this score was proven in our study, the NEWS2 score showed the lowest sensitivity and specificity among the evaluated scores. Certain studies were evaluating the efficacy of the initial score and maximum score in predicting poor outcomes, having in mind the unpredictability of the COVID-19 clinical course [19,20]. The risk of mortality and admission to ICU was also related to the change from baseline to maximum score, showing good predictability of the score regarding the short-term (2-day) mortality [21].

REMS (Rapid Emergency Medicine Score) is a composite score consisting of the Glasgow Coma Score (GCS), respiratory rate, oxygen saturation, mean arterial pressure (MAP), hazard ratio, and age [22]. All of the parameters are scored with grades from 0 to 4 with a maximum score of 26. This score is easy to implement into everyday clinical practice, as it combines standard parameters in evaluating patients’ conditions, without the need for certain extra procedures or laboratory parameters. It is superior in predicting in-hospital mortality and non-inferior to some other commonly used scores (APACHE-II and RASP score) [23]. Imhoff et al. validated the REMS in a retrospective study and found that a 1-point increase on the 26-point REMS scale was associated with an odds ratio of 1.40 for in-hospital death [24]. The superiority of REMS over other predictive scores might have been because of age as a component. It is shown that the analyses of patients older than 70 years demonstrated a better diagnostic and discrimination capacity of EDWs for both mortality outcomes than in younger patients [25]. This is important to underline, as age was an independent predictor of mortality in our study cohort, while REMS showed the highest sensitivity and specificity among evaluated scores (72.1 and 74.9%, respectively), with the highest negative predictive value of 96.8. Certain studies also proved the prognostic significance of this score in patients with COVID-19 [26]. Ruangsomboon et al. concluded that REMS had the highest prognostic utility as it outperformed qSOFA, MEWS, and NEWS in predicting in-hospital mortality in COVID-19 patients [27].

qSOFA (quick SOFA score) identifies high-risk patients for in-hospital mortality with suspected infection outside the ICU [28]. It incorporates systolic blood pressure, respiratory rate and GSC, and it is scored with a maximum of 3 points. A score greater than or equal to 2 represents a greater risk of a fatal outcome. Previous studies demonstrated excellent results in predicting in-hospital mortality in patients with COVID-19 [27,29]. It is shown that patients with a qSOFA score above 2 have an 11-fold higher mortality risk compared to patients with a score below 2 [28,30]. However, this score cannot be used to assume short-term stable or noncritical disease status in COVID-19, which is mainly because of its low sensitivity.

The development of COVID-19-dedicated risk scores to predict poor clinical outcomes was also an important task in several studies. Zdravkovic et al. developed a simple and effective score to predict mortality in patients admitted to ICU [31]. The score has a high discriminative value with a sensitivity of 82.4% (95% CI 76.7% to 87.1%), a specificity of 41.0%, and C statistic of 0.863. It is easy to implement into everyday clinical practice, as it includes four important parameters (age, IL-6, D-dimer, and serum albumin), reflecting the main pathophysiological mechanisms of the disease (inflammation, thromboembolism and cytokine storm).

The present study has some limitations to be taken into account. Considering that this is a single-center study, additional multicenter prospective studies are needed to validate the predictive accuracy of the evaluated scores. However, the study sample size included almost 4000 patients. The impact of vaccination is also undetermined, as the majority of the population did not finish the immunization until the end of the study period.

## 5. Conclusions

Among evaluated, widely used, early warning risk scores to predict mortality and ICU admission among COVID-19 patients, all evaluated predictive scores (MEWS, NEWS, NEWS2, REMS, qSOFA) showed significant diagnostic performance, with REMS being the most sensitive and specific. Larger, multicenter studies are needed to provide definitive evidence of the prognostic value of these scores in everyday clinical practice. Having in mind the variations in diagnostic performance of already derived early warning scores, the development of new, COVID-19 dedicated risk scores is mandatory. This can provide more sufficient risk stratification, less consumption of health care resources, and better clinical outcomes not only in terms of COVID-19 disease but also in terms of other diseases with global impact.

## Figures and Tables

**Table 1:** Sociodemographic characteristics and comorbidities of the study population according to outcome.

Variable	Outcome		*p*
	Survival (*n* = 3311)	Exitus Letalis (*n* = 299)	
Gender, *n* (%)			
Male	2075 (62.7)	169 (56.5)	**0.035**
Female	1234 (37.3)	130 (43.5)	
Age, mean ± sd	57.9 ± 14.9	73.4 ± 11.7	**<0.001**
Comorbidities, *n* (%)	1888 (57.1)	230 (76.9)	**<0.001**
Hypertension	1564 (47.3)	177 (59.2)	**<0.001**
Diabetes mellitus	559 (16.9)	95 (31.8)	**<0.001**
Obesities	188 (5.7)	21 (7.0)	0.334
COPD	89 (2.7)	17 (5.7)	**0.003**
Asthma	121 (3.7)	10 (3.3)	0.782
Coronary disease	276 (8.3)	62 (20.7)	**<0.001**
Cardiomyopathy	148 (4.5)	51 (17.1)	**<0.001**
Malignancy	149 (4.5)	42 (14.0)	**<0.001**

**Table 2:** Radiographic findings, need for oxygen support, hospital-acquired pneumonia and superinfection, ARDS and therapy according to outcome.

Variable	Outcome		*p*
	Survival (*n* = 3311)	Exitus Letalis (*n* = 299)	
Radiographic findings, *n* (%)			
Normal/Unilateral pneumonia	875 (26.8)	56 (19.3)	**0.005**
Bilateral pneumonia	2388 (73.2)	234 (80.7)	
CT score, median (25–75th percentile)	10 (7–14)	17 (11–22)	**<0.001**
Admission to ICU, yes, *n* (%)	166 (5.0)	197 (65.9)	**<0.001**
Need for oxygen support, *n* (%)			
O_2_ mask/HFNC	3235 (97.9)	264 (88.6)	**<0.001**
NIV/IMV	70 (2.1)	34 (11.4)	
Hospital-acquired pneumonia, yes, *n* (%)	53 (1.6)	68 (22.7)	**<0.001**
Healthcare-associated pneumonia	34 (70.8)	21 (31.8)	**<0.001**
Ventilator-associated pneumonia	14 (29.2)	45 (68.2)	
Bacterial superinfection, yes, *n* (%)	111 (3.4)	121 (40.5)	**<0.001**
ARDS, yes, *n* (%)	31 (1.1)	204 (70.3)	**<0.001**
Corticosteroid therapy, yes, *n* (%)	2372 (71.7)	250 (83.6)	**<0.001**

**Table 3:** Laboratory parameters of the study population according to outcome.

Variable	Outcome		*p*
Median (25–75th Percentile)	Survival (*n* = 3311)	Exitus Letalis (*n* = 299)	
Erythrocytes	4.68 (4.33–5.03)	4.37 (3.86–4.82)	**<0.001**
Hemoglobin	138 (127–148)	128.5 (111–140)	**<0.001**
Hematocrit	0.41 (0.38–0.44)	0.39 (0.34–0.42)	**<0.001**
Leukocytes	5.38 (4.52–7.71)	7.57 (5.38–11.64)	**<0.001**
Neutrophils	3.65 (2.27–5.35)	5.71 (3.23–9.66)	**<0.001**
Lymphocytes	1.22 (0.85–1.86)	0.83 (0.57–1.28)	**<0.001**
Neutrophils/Lymphocytes ratio	3.11 (1.68–5.33)	6.57 (3.39–13.5)	**<0.001**
Thrombocytes	199 (158–255)	189 (136–266)	**0.021**
Il-6	23.9 (10.2–52.01)	81.1 (38.35–173.35)	**<0.001**
INR	1.04 (0.97–1.13)	1.13 (1.02–1.33)	**<0.001**
aPTT	24.2 (22.6–26.1)	25.6 (23.1–29.1)	**<0.001**
Fibrinogen	4.0 (3.4–5.0)	4.2 (3.4–5.3)	**0.041**
D-dimer	440 (253–820)	1125 (520–3022)	**<0.001**
PCT	0.08 (0.05–0.15)	0.27 (0.15–0.76)	**<0.001**
CRP	34.7 (10.4–78.2)	85.7 (43.1–168.4)	**<0.001**
Urea	5.4 (4.3–7.1)	9.3 (6.3–14.6)	**<0.001**
Creatinine	88 (75–105)	108 (83–147)	**<0.001**
Glycose	6.3 (5.6–7.4)	7.6 (6.3–10.1)	**<0.001**
AST	29 (22–42)	38 (27–57)	**<0.001**
ALT	27 (18–43)	24 (17–42)	0.091
Bilirubin	8.4 (6.1–11.8)	9.6 (6.7–15.3)	**<0.001**
LDH	454 (358–601)	650 (461–875)	**<0.001**
Ferritin	440 (243.5–754)	689 (371–1194)	**<0.001**

**Table 4:** Glasgow Coma Score, pulmonary and hemodynamic parameters of the study population according to outcome.

Variable	Outcome		*p*
	Survival (*n* = 3311)	Exitus Letalis (*n* = 299)	
Glasgow Coma Score, *n* (%)			
=15	3231 (97.6)	234 (79.1)	**<0.001**
<15	79 (2.4)	62 (20.9)	
FiO_2_ at admission, *n* (%)			
=21	3260 (98.5)	286 (95.7)	**<0.001**
>21	51 (1.5)	13 (4.3)	
Number of respirations, median (25–75th percentile)	14 (14–15)	16 (14–18)	**<0.001**
SpO_2_, mean ± sd	95.2 ± 4.3	89.6 ± 9.7	**<0.001**
Systolic blood pressure (mm/Hg), mean ± sd	127.1 ± 16.8	125.3 ± 20.6	0.089
Diastolic blood pressure (mm/Hg), mean ± sd	79.4 ± 10.8	76.0 ± 12.7	**<0.001**
MAP	95.2 ± 11.6	92.4 ± 14.1	**<0.001**
Heart rate (/min), mean ± sd	82.9 ± 13.9	87.3 ± 19.4	**<0.001**

**Table 5:** Prediction scores of the study population according to outcome.

Variable	Outcome		*p*
	Survival (*n* = 3311)	Exitus Letalis (*n* = 299)	
MEWS			
0	1813 (54.8)	90 (30.2)	**<0.001**
>0	1497 (45.2)	208 (69.8)	
NEWS, median (25–75th percentile)	3 (2–4)	5 (3–7)	**<0.001**
NEWS2, median (25–75th percentile)	3 (2–3)	3 (2–5)	**<0.001**
REMS			
≤5	2480 (74.9)	83 (27.9)	**<0.001**
>5	830 (25.1)	215 (72.1)	
qSOFA			
0	3014 (91.1)	197 (66.1)	**<0.001**
>0	296 (8.9)	101 (33.9)	

**Table 6:** Measures of diagnostics accuracy of prediction scores.

Variable	Outcome			
	Sensitivity	PPV	Specificity	NPV
MEWS	69.8	12.2	54.8	95.3
NEWS	70.6	13.4	58.6	95.7
NEWS2	61.9	8.9	42.8	92.6
REMS	72.1	20.6	74.9	96.8
qSOFA	33.9	25.4	91.1	93.9

**Table 7:** Multivariate logistic regression analysis with outcome as a dependent variable.

Variable	Multivariate		
	*p*	OR	95% CI for OR
Age	**<0.001**	1.056	1.032–1.081
CT Score	**<0.001**	1.135	1.096–1.174
Urea	**<0.001**	1.082	1.038–1.128
Bacterial superinfection	**<0.001**	13.554	8.741–21.017
REMS	**0.002**	1.183	1.063–1.316

## Data Availability

The data that support the findings of this study are available from the corresponding author (MZ) upon reasonable request.

## Supplementary Materials

The following supporting information can be downloaded at: https://www.mdpi.com/article/10.3390/diagnostics13162653/s1, Supplemental Table S1. Scoring and interpretation of evaluated Early Warning Scores (EWS).
