# Supplementary material for: The Predictive Value of Risk Factors and Prognostic Scores in Hospitalized COVID-19 Patients
Source: Diagnostics (Basel). 2023 Aug 11;13(16):2653. doi: 10.3390/diagnostics13162653 (PMC10453362; doi:10.3390/diagnostics13162653)
Supplement: Supplementary file 1 [file diagnostics-13-02653-s001.zip › diagnostics-2501023-supplementary.pdf]

MEWS

SYSTOLIC BP		HEART RATE		RESPIRATORY RATE		TEMPERATURE		APVU SCORE	
≤70 mmHg	+3	<40 bpm	+2	<9 bpm	+2	<35°C / 95°F	+2	Alert	0
71-80 mmHg	+2	41-50bpm	+1	9-14 bpm	0	35–38.4°C / 95–101.1°F	0	Reacts to voice	+1
81-100mm Hg	+1	51-100bpm	0	15-20bpm	+1	≥38.5°C / 101.3°F	+2	Reacts to pain	+2
101-199m mHg	0	101-110bpm	+1	21-29bpm	+2			Unresponsive	+3
≥200 mmHg	+2	111-129bpm	+2	≥30 bpm	+3				
		≥130 bpm	+3						

- A score ≥5 is statistically linked to increased likelihood of death or admission to an intensive care unit.
- For any single physiological parameter scored +3, consider higher level of care for patient.

NEWS

SYSTOLIC BP		HEART RATE		RESPIRATORY RATE		TEMPERATURE		APVU SCORE		SaO2		SUPPLEMENT. O2	
≤90 mmHg	+3	<40 bpm	+3	<8 bpm	+3	≤35°C / 95°F	+3	Alert	0	≤91 %	+3	YES	+2
91-100 mmHg	+2	41-50 bpm	+1	9-11 bpm	+1	35.1-36°C / 95.1-96.8°F	+1	Voice Pain Unresponsive	+3	92-93 %	+2	NO	0
101-110 mmHg	+1	51-90 bpm	0	12-20 bpm	0	36.1-38°C / 96.9-100.4°F	0			94-95 %	+1		
111-219 mmHg	0	91-110 bpm	+1	21-24 bpm	+2	38.1-39°C / 100.5-102.2°F	+1			≥96 %	0		
≥220 mmHg	+3	111-130 bpm	+2	≥25 bpm	+3	≥39.1 °C / 102.3°F	+2						
		≥131 bpm	+3										

- A low score (NEWS 1–4) should prompt assessment by a competent registered nurse who should decide if a change to frequency of clinical monitoring or an escalation of clinical care is required.
- A medium score (ie NEWS of 5–6 or a RED score) should prompt an urgent review by a clinician skilled with competencies in the assessment of acute illness – usually a ward-based doctor or acute team nurse, who should consider whether escalation of care to a team with critical-care skills is required (ie critical care outreach team). A RED score refers to an extreme variation in a single physiological parameter (i.e., a score of 3 on the NEWS chart in any one physiological parameter, colored RED to aid identification; e.g., heart rate
- A high score (NEWS ≥7) should prompt emergency assessment by a clinical team/ critical care outreach team with critical-care competencies and usually transfer of the patient to a higher dependency care area.

NEWS2

SYSTOLIC BP		HEART RATE		RESPIRATORY RATE		TEMPERATURE		APVU SCORE		HYPERCAPNIC RESP. FAILURE				SUPPLEMENT. O2	
≤90 mmHg	+3	<40 bpm	+3	<8 bpm	+3	≤35°C / 95°F	+3	Alert	0	NO		YES		YES	+2
91-100 mmHg	+2	41-50 bpm	+1	9-11 bpm	+1	35.1-36°C / 95.1-96.8°F	+1	Voice Pain Unresponsive	+3	≤91%	+3	≤83%	+3	NO	0
101-110 mmHg	+1	51-90 bpm	0	12-20 bpm	0	36.1-38°C / 96.9-100.4°F	0			92-93 %	+2	84-85%	+2		
111-219 mmHg	0	91-110 bpm	+1	21-24 bpm	+2	38.1-39°C / 100.5-102.2°F	+1			94-95 %	+1	86-87%	+1		
≥220 mmHg	+3	111-130 bpm	+2	≥25 bpm	+3	≥39.1 °C / 102.3°F	+2			≥96%	0	88-92%, ≥93% on room air	0		
		≥131 bpm	+3									93-94% on supplemental O_2	+1		
												95-96% on supplemental O_2	+2		
												≥97% on supplemental O_2	+3		

- Score 0-4: Clinical Risc Low,Frequency of monitoring every 12 hours if score is 0 and every 6 hours if score is 1-4. (Assessment by a competent registered nurse or equivalent, to decide change in frequency of clinical monitoring or escalation of care)
- Score of 3 in any individual parameter: Low-medium clinical risc; monitoring every hour; (Urgent review by a ward-based doctor, to decide change in frequency of clinical monitoring or escalation of care)
- Score 5-6: Medium clinical risc; monitoring every hour minimum; (Urgent review by a ward-based doctor or acute team nurse, to decide if critical care team assessment is needed)

- Score ≥7: High clinical risc; Continuous monitoring of vital signs; (Emergent assessment by a clinical team or critical care team and usually transfer to higher level of care)

REMS

AGE		MAP		HEART RATE		RESPIRATORY RATE		SaO2		GCS	
<45	0	>159mmHg	+4	>179bpm	+4	49bpm	+4	<75%	+4	<5	+4
45-54	+2	130-159mmHg	+3	140-179bpm	+3	35-49bpm	+3	75-85%	+3	5-7	+3
55-64	+3	110-129mmHg	+2	110-139bpm	+2	25-34bpm	+1	86-89%	+1	8-10	+2
65-74	+5	70-109mmHg	0	70-109bpm	0	12-24bpm	0	>89%	0	11-13	+1
>74	+6	50-69mmHg	+2	55-69bpm	+2	10-11bpm	+1			>13	0
		≤49mmHg	+4	40-54bpm	+3	6-9bpm	+2				
				≤39bpm	+4	≤5bpm	+4				

Interpretation:

REMS	In-hospital mortality
0-2	0%
3-5	1%
6-9	3%
10-11	4%
12-13	10%
14-15	17%
16-17	38%
18-19	75%
20-21	56%
22-23	66%
24-26	100%

qSOFA

Altered mental status		Respiratory rate ≥22		Systolic blood pressure ≤100	
NO	0	NO	0	NO	0
YES	+1	YES	+1	YES	+1

Score 0-1 : Not high risk for in-hospital mortality
Score 2-3: High risk for in-hospital mortality (3- to 14-fold increase)
